# Gene expression ranking change based single sample pre-disease state detection

**DOI:** 10.3389/fgene.2024.1509769

**Published:** 2024-12-04

**Authors:** Zhenshen Bao, Xianbin Li, Peng Xu, Xiangzhen Zan

**Affiliations:** ^1^ School of Information Engineering, Taizhou University, Taizhou, Jiangsu, China; ^2^ School of Computer and Big Data Science, Jiujiang University, Jiujiang, Jiangxi, China; ^3^ Institute of computational science and technology, Guangzhou University, Guangzhou, Guangdong, China; ^4^ School of Cultural and Creative Trade, Shenzhen Pengcheng Technician College, Shenzhen, Guangdong, China

**Keywords:** pre-disease state, state transition, single sample, ranking change, personalized disease diagnosis

## Abstract

**Introduction:**

To prevent disease, it is of great importance to detect the critical point (pre-disease state) when the biological system abruptly transforms from normal to disease state. However, rapid and accurate pre-disease state detection is still a challenge when there is only a single sample available. The state transition of the biological system is driven by the variation in regulations between genes.

**Methods:**

In this study, we propose a rapid single-sample pre-disease state-identifying method based on the change in gene expression ranking, which can reflect the coordinated shifts between genes, that is, S-PCR. The R codes of S-PCR can be accessed at https://github.com/ZhenshenBao/S-PCR.

**Results:**

This model-free method is validated by the successful identification of pre-disease state for both simulated and five real datasets. The functional analyses of the pre-disease state-related genes identified by S-PCR also demonstrate the effectiveness of this computational approach. Furthermore, the time efficiency of S-PCR is much better than that of its peers.

**Discussion:**

Hence, the proposed S-PCR approach holds immense potential for clinical applications in personalized disease diagnosis.

## Introduction

The biological system may undergo sudden deterioration during the progression of a complex disease (Venegas et al., 2005; McSharry et al., 2003), which conforms to the bifurcation and critical slowing down theory. Based on this feature, Chen et al. divvied the progression of complex disease into three states: a normal state, a disease state, and a pre-disease state (Chen et al., 2012; Liu et al., 2012). When the system is in the normal state, it is stable, under control, and with high resilience. When the system is in the disease state, it is also stable but in deterioration. When the system is in the pre-disease state, it is unstable and easily revised to the normal state with appropriate intervention but may transform into the deteriorated disease state without intervention. Thus, it is imperative to identify such pre-disease states to signal the upcoming disease and take suitable interventions to prevent the disease.

High-throughput technologies enable us to observe the expression of thousands of genes in a single sample at the same time, which makes it possible to measure the long-term dynamics of the biological system. However, the pre-disease state is defined as the limit of the normal state (Achiron et al., 2010); thus, the mean gene expression of the pre-disease state is similar to that of the normal state. Hence, it is a challenge to accurately detect the pre-disease state as the early warning signal for the disease state *via* time series gene expression data. To solve this problem, Chen et al. proposed a model-free method called the dynamic network biomarker (DNB), which selects a small group of genes using three statistical measurements: the average Pearson’s correlation coefficients (PCCs) of these genes significantly increase, the average PCCs of these genes between this group and any other significantly decrease, and the average standard deviations (SDs) of these selected genes significantly increase (Chen et al., 2012; Liu et al., 2012). The implementation of these measurements needs multiple measures for an individual. However, clinical experiments can usually provide only a single sample for an individual, which limits the clinical application of the DNB approach. Therefore, there is an urgent requirement to develop methods to identify the pre-disease state as early warning signals for disease using the clinical single-sample dataset. Recently, inspired by the DNB method, many single-sample suitable methods have been proposed. These methods can be divvied into two classes. The first category of methods is mainly based on the network and entropy, for example, DNB-s (Liu et al., 2014), iENA (Yu et al., 2017), SLE (Liu et al., 2020), SNE (Han et al., 2020), SSP (Huo et al., 2022), DNRS (Zhong et al., 2022), SNPE (Zhong et al., 2023), and ERE (Hong et al., 2024; Bao et al., 2022; Huo et al., 2023). The second category of methods is gene expression distribution-based methods, which focus on gene expression distribution differences between normal and pre-disease states, such as sKLD (Zhong et al., 2020) and sJSD (Yan et al., 2021). These methods have successfully identified the pre-disease state. However, due to the application of complex biological networks and the distribution comparison for thousands of genes, the application of these methods remains limited. The running time of such a method should be as less as possible for the timely treatment to reverse the state of biological systems in clinical practice. Therefore, developing a rapid and accurate method to detect the pre-disease state is still an open problem.

According to the DNB theory (Chen et al., 2012; Liu et al., 2012), the expression of DNBs highly fluctuates and is highly associated with each other at the pre-disease state. In a biological system, the huge changes in the expression rankings of two genes not only signify a significant alteration in their expression patterns but also imply coordinated shifts in their expressions, revealing a close interplay between the two genes (Subramanian et al., 2005). Thus, the genes with significant changes in expression ranking also highly interact with each other. Specifically, the genes with huge changes in their expression value and ranking can be considered DNBs, which are the outcome of the underlying system (Liu et al., 2014). In this study, we propose a rapid model-free single-sample pre-disease state-identifying method based on the change in gene expression ranking, that is, S-PCR. The results of both simulation and real datasets demonstrate that the proposed S-PCR can detect the early warning signals before the system reaches a critical transition/pre-disease state. The results of the functional analysis for the two influenza virus infections and three cancer datasets indicate that the genes selected by S-PCR are highly associated with the development of diseases. Furthermore, benefitting from gene expression ranking while accurately detecting the pre-disease state, S-PCR achieves faster computational speed than other methods. Hence, the proposed approach holds immense potential for clinical applications as it enables the fast identification of individual-specific states from a single sample, thereby facilitating personalized disease prediction.

## Materials and methods

### Algorithm to detect the pre-disease state using S-PCR

**FIGURE 1:**
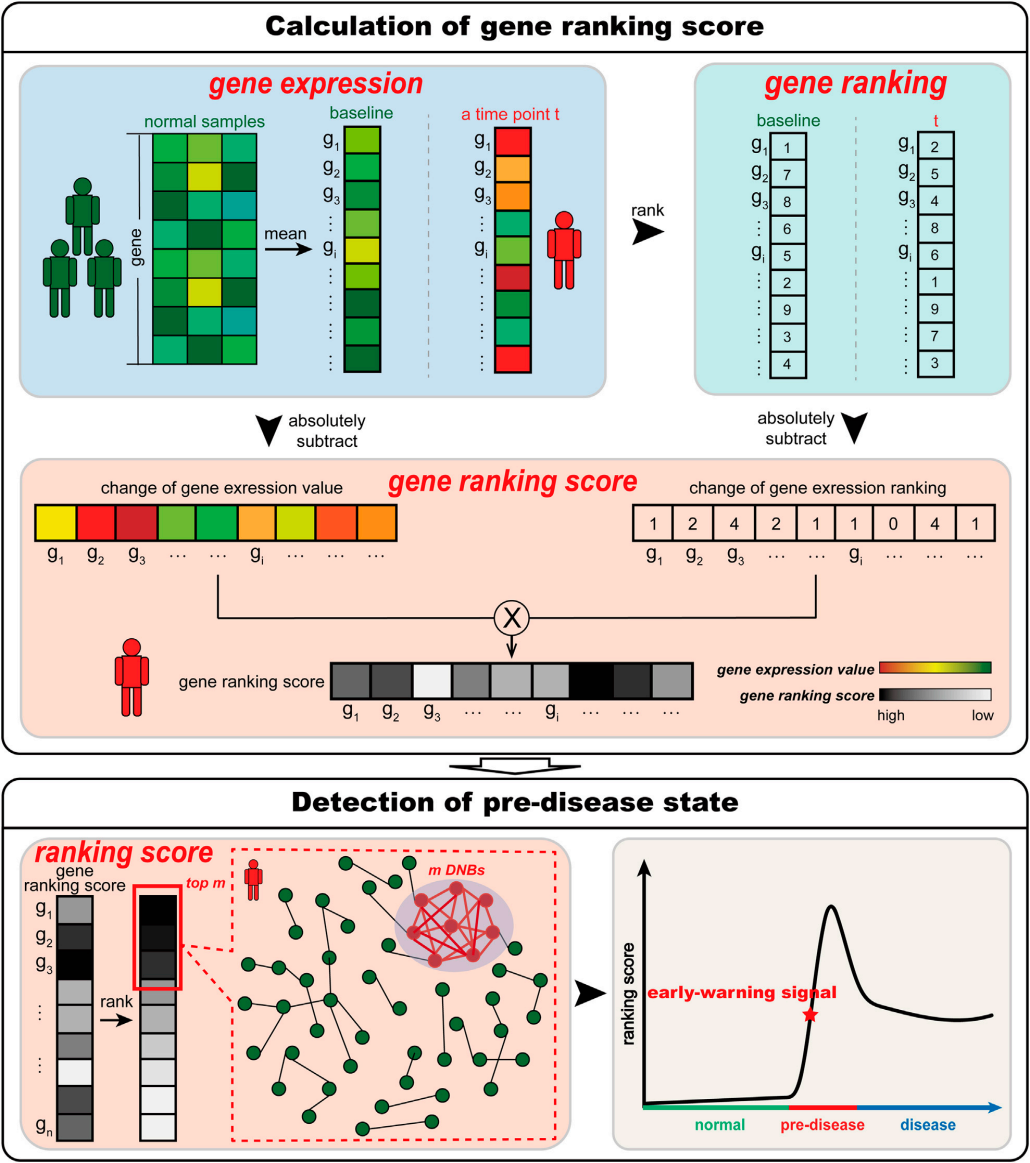
Schematic illustration of S-PCR. Given a number of normal samples to calculate the baseline data, S-PCR is calculated based on a single sample from any individual. Specifically, the ranking of all genes for both the baseline sample and the to-be-determined single sample is calculated. For each gene, the local ranking score is calculated by combining the expression and ranking changes. Given the time-course samples from an individual, the early warning signal for the pre-disease state can be detected through the significant increase in the ranking score.

According to the DNB theory (Chen et al., 2012; Liu et al., 2012), when the system is close to the pre-disease state, there is a group of highly related genes (DNBs) whose expressions widely fluctuate. Thus, to determine whether the sample of a time point is in the pre-disease state, we should identify whether such a set of DNBs exists at this time point. For a time point, S-PCR combines the change in the expression value and ranking. The change in the gene expression value used in S-PCR is to identify expression fluctuating signals of DNBs. The change in gene expression ranking is used to amplify such signals and seek the set of DNBs. The pre-disease state in S-PCR is detected by the following procedures (see Figure 1).

Given *n* normal samples which are all in the normal state, the expression value $E_{i}^{b}$ of a gene $g_{i}$ at the time point *baseline* is calculated as Formula 1.

$$E_{i}^{b}=mean\left(E_{i}^{1},E_{i}^{2},\cdots ,E_{i}^{n}\right),$$ (1)

where $E_{i}^{1},E_{i}^{2},\cdots ,E_{i}^{n}$ are the expression values of gene $g_{i}$ in the *n* normal samples.

For a time point *t*, the expression change $\Delta E_{i}^{t}$ of a gene $g_{i}$ is calculated as Formula 2.

$$\Delta E_{i}^{t}=\left|E_{i}^{t}-E_{i}^{b}\right|,$$ (2)

where $E_{i}^{t}$ and $E_{i}^{b}$ are the expression values of gene $g_{i}$ at the time point *t* and baseline, respectively. Then, the expression ranking change $\Delta R_{i}^{t}$ of that gene at the time point *t* is calculated as Formula 3.

$$\Delta R_{i}^{t}=\left|R_{i}^{t}-R_{i}^{b}\right|,$$ (3)

where $R_{i}^{t}$ and $R_{i}^{b}$ are the expression ranking of gene $g_{i}$ at the time point *t* and baseline, respectively. The local score $S_{i}^{t}$ of gene $g_{i}$ at the time point *t* can be gained by combining the expression change $\Delta E_{i}^{t}$ and expression ranking change $\Delta R_{i}^{t}$ , as Formula 4 shown.

$$S_{i}^{t}=\Delta E_{i}^{t}\cdot \Delta R_{i}^{t}.$$ (4)

The genes with coordinated shifts in their expressions may have close interplays (Subramanian et al., 2005). Furthermore, the DNBs are always a little number of genes with huge change in their expressions and closely interact with each other (Chen et al., 2012; Liu et al., 2012). Thus, the system state at time point *t* can be represented as the score $S^{t}$ , which is calculated based on the top *m* (*m* = 50) genes with the largest score $S_{i}^{t}$ , as Formula 5 shown.

$$S^{t}=\frac{1}{m}\sum_{i=1}^{m}\frac{S_{i}^{t}}{N},$$ (5)

where *N* is the total number of genes measured for each time point of a single sample. To better seek the early warning signal for the pre-disease state, the ranking score $\Delta S^{t}$ is calculated from the score $S^{t}$ for the time point *t*, as Formula 6 shown.

$$\Delta S^{t}=\left|S^{t}-S^{0}\right|,$$ (6)

where $S^{0}$ is the ranking score of the initial time point, which is the first time point after the *baseline*.

When the system is near the pre-disease state, the two measures $\Delta E_{i}^{t}$ and $\Delta R_{i}^{t}$ of these DNB biomolecules will suddenly increase. Then, the ranking score $\Delta S^{t}$ will sharply increase accordingly. Thus, the significant increase in the ranking scores for an individual can be considered an early warning signal for the pre-disease state and help understand the significantly collective fluctuating behaviors of genes in the pre-disease state.

### Determination of the threshold for the detection of the pre-disease state

To identify the pre-disease state, the determination of the threshold θ for the ranking scores $\Delta S^{t}$ plays an important role. The threshold θ is calculated from the scores of normal samples, as follows:

Step 1: calculating the ranking scores $\Delta S^{t}$ for all normal samples.
Step 2: normalizing the ranking scores of all normal samples in a dataset to z-scores using the mean and standard deviation of these ranking scores.
Step 3: assuming that these z-scores follow a standard normal distribution, the value corresponding to the upper 0.05 is set as the threshold θ of the ranking scores for detecting the pre-disease state.

### Data processing and functional analysis

**FIGURE 2:**
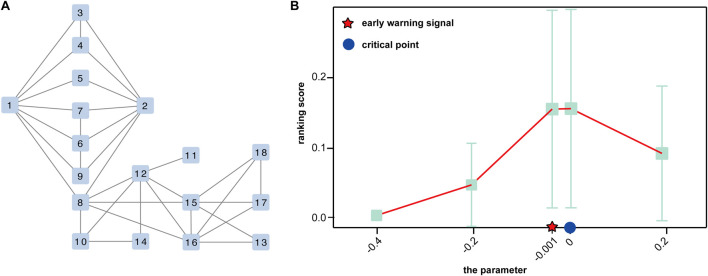
Performance of the proposed method of the simulated dataset. **(A)** A 18-node regulatory network. **(B)** Curve of the ranking score.

To show the utility of S-PCR, a simulated gene regulatory network with 18 genes was used, as shown in Figure 2A. Such a network follows the Michaelis–Menten form and is a classical model for studying non-linear biological processes (Srinivasan, 2022). A detailed description of the network characterized by a set of 18 stochastic differential equations in Michaelis–Menten form was provided by Zhong et al. (2022). Then, the numeric simulation dataset was generated from the network based on a parameter *q* varying from −0.4 to 0.2 and *q* = 0 as the critical point (Zhong et al., 2022).

Two real-time course gene expression datasets are obtained from the GEO database (https://www.ncbi.nlm.nih.gov/geo/), including influenza virus H3N2 infection dataset GSE30550 and H1N1 infection dataset GSE52428 (Huang et al., 2011; Woods et al., 2013). Each gene is mapped by multiple probes, and the average value of these probes is used as the gene expression. The probes without the corresponding NCBI Entrez gene symbol are discarded. Three tumor disease datasets from the TCGA database (http://cancergenome.nih.gov), namely, breast cancer (BRCA), esophageal carcinoma (ESCA), and rectum adenocarcinoma (READ), are composed of both tumor and tumor-adjacent samples. The tumor samples are grouped into different stages according to the stage information of TCGA, and the samples lacking corresponding information are ignored. Table 1 lists the detailed information of the five datasets.

**TABLE 1:** Details of the real datasets.

Dataset	Hour/stage	Subject
GSE30550	Baseline, 0, 5, 12, 21, 29, 36, 45, 53, 60, 69, 77, 84, 93, 101, and 108	Sx:9/Asx:8
GSE52428	Baseline, 0, 5, 12, 21.5, 29, 36, 45.5, 53, 60, 69.5, 77, 84, 93.5, 101, and 108	Sx:9/Asx:15
BRCA	I, IA, IB, II, IIA, IIB, III, IIIA, IIIB, IIIC, IV, and X	-
ESCA	I, IA, IB, II, IIA, IIB, III, IIIA, IIIB, IIIC, IV, IVA, and IVB	-
READ	I, II, IIA, IIB, IIC, III, IIIA, IIIB, IIIC, IV, and IVA	-

Sx, symptomatic subjects; Asx, asymptomatic subjects.

For the five real datasets, the top 5% genes with the largest ranking scores at the early warning signal appearing the time point are selected as candidate signaling genes. Functional analyses are carried out using GO term enrichment and KEGG pathway analysis based on these genes to find the early biological features of diseases. The GO term enrichment analysis is carried out using the web analysis tool DAVID (https://david.ncifcrf.gov/) (Sherman et al., 2022; Huang da et al., 2009). KEGG pathway analysis is performed using the key pathways in the Kyoto Encyclopedia of Genes and Genomes database (https://www.kegg.jp) (Kanehisa et al., 2024). Gene analysis is performed based on the GeneCards database (https://www.genecards.org/).

## Results

The theoretical background and computational algorithm of S-PCR were delineated in the preceding section. To demonstrate the effectiveness of S-PCR, we implemented it on a simulated dataset, two influenza infection datasets, and three cancer datasets. The detailed description of these datasets is provided in the previous section. For all datasets, the proposed S-PCR successfully detected the early warning signals for critical points, which validated the effectiveness of our method in quantifying critical points just before critical transitions into irreversible disease states. In this process, some genes with high ranking scores in the pre-disease state were selected as candidate signaling genes for further analysis. To further validate the performance of our method, we compared it with the following peer methods: SLE (Liu et al., 2020) and sJSD (Yan et al., 2021), which are the representative methods in the two classes of pre-disease state prediction. In addition, the function of the expression ranking change was validated in the following result.

### Validation based on numerical simulation

The numerical simulation dataset generated from a classical 18-node regulatory network (Figure 2A), which was represented in the Michaelis–Menten form, is used to demonstrate the performance of the proposed method (Zhong et al., 2022). In the dataset, a special parametric value, *q* = 0, is the critical point of the dynamic system.

As shown in Figure 2B, the ranking score increases rapidly when the regulatory network model arrives at a special parametric value, *q* = 0, which is the critical point of the dynamic system. Moreover, there is a sharp increase at a parameter value *q* = −0.001, indicating the upcoming critical point at the parameter value *q* = 0. Such a signal can be successfully detected by our method, which demonstrates the effectiveness of S-PCR in detecting the early warning signal of the pre-disease state.

### Pre-disease state identification for influenza infection

**FIGURE 3:**
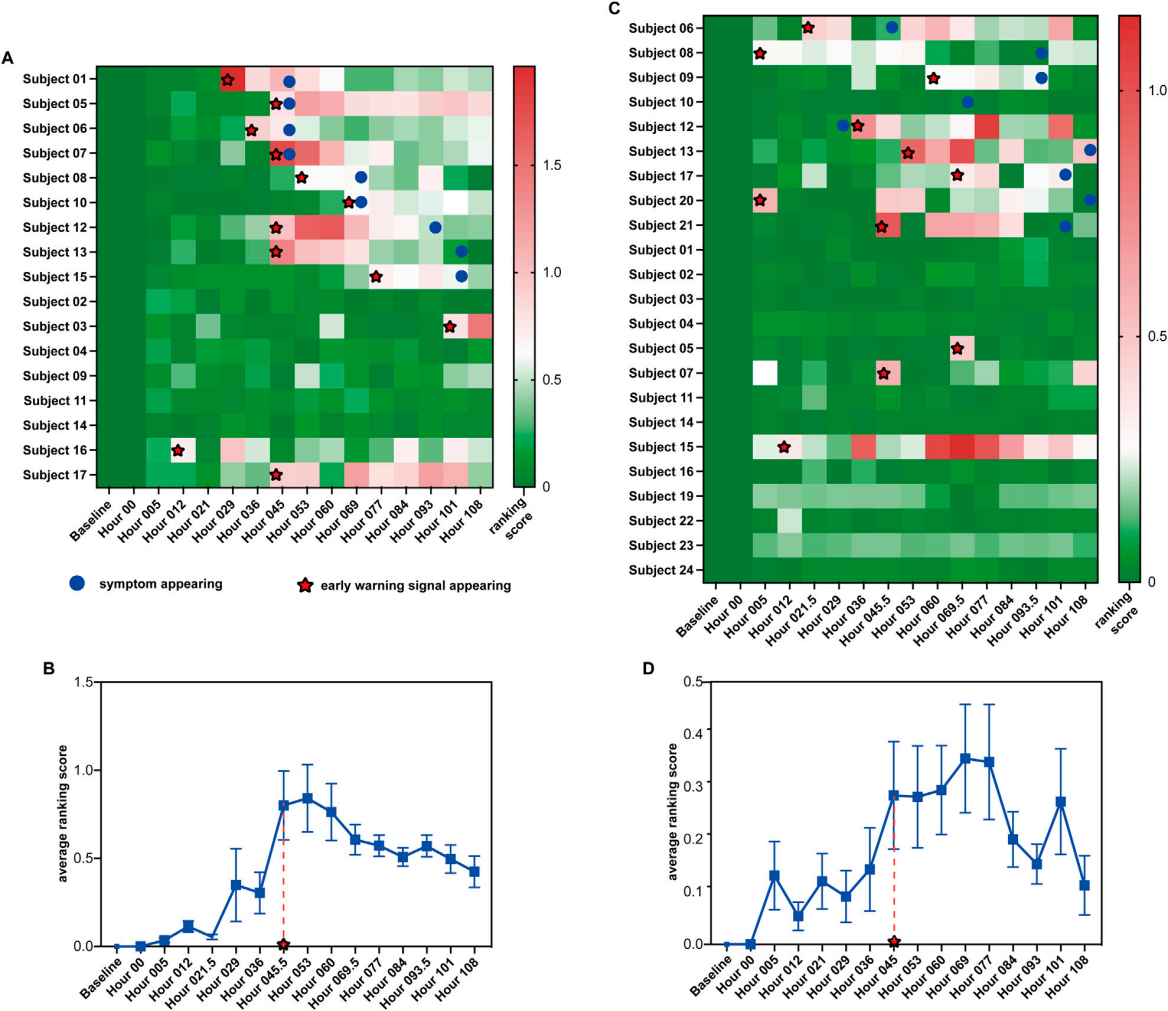
Summarized prediction results of the two influenza virus infection datasets. **(A, C)** Heatmap of prediction results for datasets GSE30550 and GSE52428. **(B, D)** Average ranking scores across all symptomatic subjects from datasets GSE30550 and GSE52428.

To illustrate how S-PCR works, the proposed method was applied to two time-series influenza infections, namely, GSE30550 and GSE52428. In datasets GSE30550 and GSE52428, the data were collected from 17 and 23 human adult subjects, and their influenza virus H3N2 or H1N1 infection process was recorded (Huang et al., 2011; Woods et al., 2013), respectively. For each dataset, nine subjects developed clinical symptoms of influenza infection and were called symptomatic subjects. Others did not have clinical symptoms at all time points and were called asymptomatic subjects. For each subject, the data at the time point baseline were used to calculate the data of their time point *baseline*. Following the algorithm of S-PCR in Section 2, 17 and 23 ranking scores were calculated based on all subjects at each time point for each dataset (Figures 3A, C). The samples from all asymptomatic subjects were utilized as normal samples to determine the threshold for detecting the pre-disease state. A ranking score above the threshold indicates the imminent appearance of influenza infection symptoms.

Figure 3A shows the ranking scores of the 17 H3N2 virus-infected subjects across the 16 time points in GSE30550 ( θ = 0.619). As shown in the figure, for each symptomatic subject, an early warning signal can be detected. All the signals are no later than the influenza infection diagnoses through the standardized symptom scoring record. Furthermore, six of nine signals are earlier than the symptom appearing; three of nine signals are detected at the same time points with the symptom appearance time. For five of eight asymptomatic subjects, no early warning signal can be detected. Furthermore, the ranking scores of the eight asymptomatic subjects are much more stable than those of the symptomatic subjects. In addition, for asymptomatic subjects 3, 16, and 17, early warning signals can be detected, which may indicate that the three subjects have symptoms after the experiment ended. Figure 3B shows the average ranking scores across the nine symptomatic subjects. According to the average score across 16 time points, we can also predict the average time points of the early warning signal: hour 045, which is all no later than the symptom appearance time point of each symptomatic subject.

Figure 3C shows the ranking scores of the 23 H1N1 virus-infected subjects across the 16 time points in GSE52428 ( θ = 0.2). As shown in the figure, for eight of nine symptomatic subjects, the early warning signals can be successfully detected; seven early warning signals of symptomatic subjects detected by the proposed method are earlier than the symptom appearance. Only in 3 of 15 asymptomatic subjects could early warning signals be detected. The ranking scores of these asymptomatic subjects are also more stable than those of symptomatic subjects. For symptomatic subject 10, no signal can be detected by our method. This may be due to the poor data quality of the dataset. For asymptomatic subject 07, a signal can be detected because in the clinical experiment, this subject has clinical symptoms, and the total symptom score of 5 days of this subject is larger than that of some symptomatic subjects (Woods et al., 2013). For asymptomatic subjects 05 and 10, the signals can be detected, which may be because the noise exists in the gene expression data at the time point baseline. Figure 3D shows the average ranking scores across the nine symptomatic subjects. According to the average score across 16 time points, we can also predict the average time points of the early warning signal, hour 045.5, which is earlier than 53 h detected by CCs (Li et al., 2015), and the average time point of symptom onset at 61.3 h (Woods et al., 2013).

The successful detection of early warning signals for the pre-disease state in datasets GSE30550 and GSE52428 by S-PCR demonstrates the effectiveness of this method in detecting the early warning signal of critical transition.

### Pre-disease state identification for cancers

**FIGURE 4:**
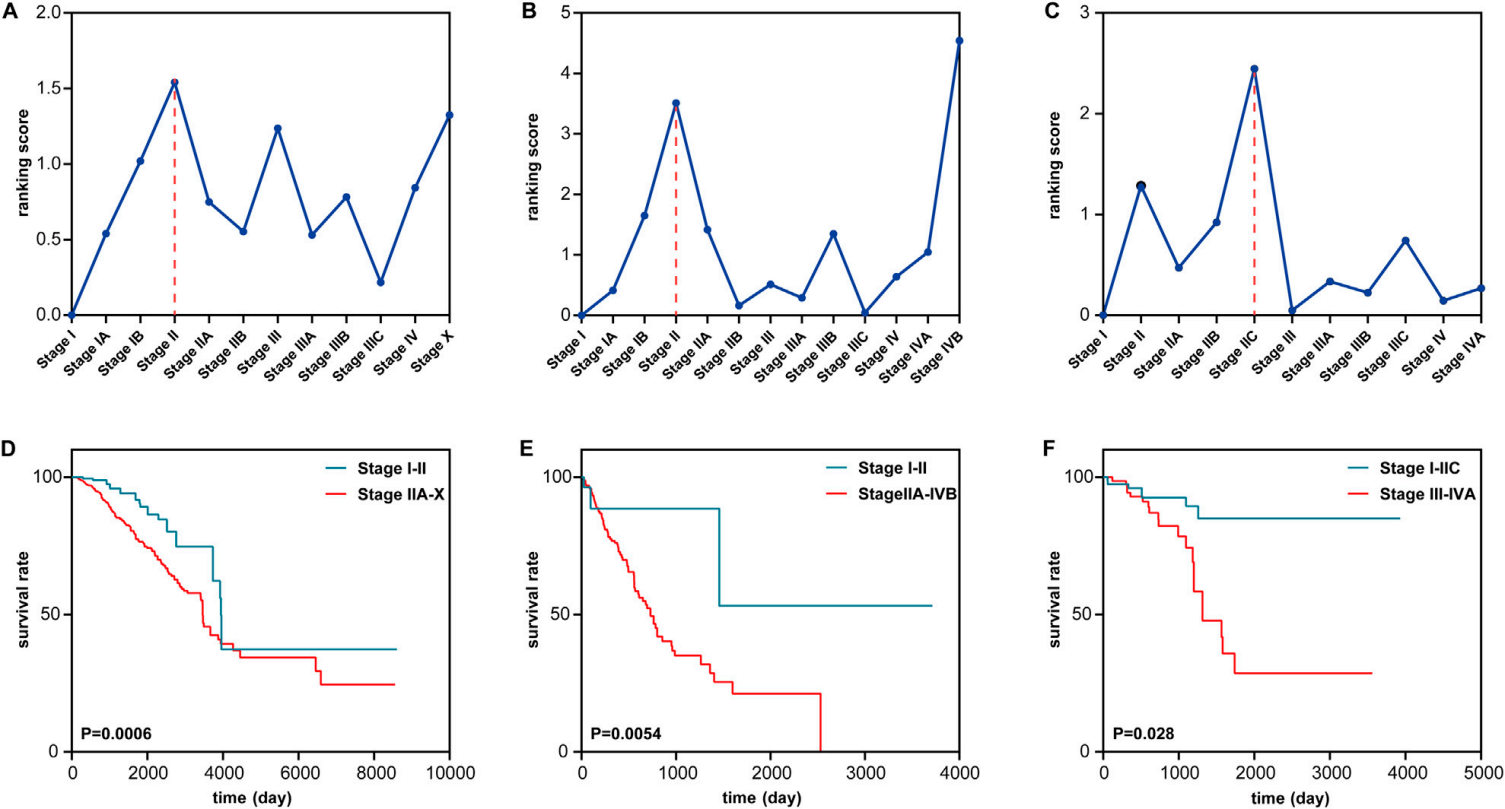
Identification of the pre-disease state of tumor near metastasis in three cancers. **(A–C)** Identifying the pre-disease state for **(A)** BRCA, **(B)** ESCA, and **(C)** READ. **(D–F)** Comparing survival curves between the before the pre-disease state and after the pre-disease state in **(D)** BRCA, **(E)** ESCA, and **(F)** READ.

To further validate the effectiveness of the proposed method, the method is applied to three tumor datasets (BRCA, ESCA, and READ) from TCGA. For each dataset, the expressions of every gene are obtained by their average value across all samples at a stage. In this way, each tumor dataset can be transformed into a single-sample dataset with only one sample for each stage. For each tumor disease dataset, the tumor-adjacent samples are utilized as reference samples to calculate the data of the time point *baseline*. However, the number of tumor-adjacent samples in the three tumor disease datasets is small, and the gene expression data from such a tumor-adjacent sample always mix with some biological noise. Thus, a ranking score threshold is not calculated for each tumor disease dataset. The pre-disease states for the three datasets are identified through the drastic increase in the ranking scores on a continuous basis after stage I. The pre-disease states of the three datasets are identified at stage II for BRCA and ESCA and at stage IIC for READ (Figures 4A–C). Metastasis is the cause behind most cancer-related deaths and the ultimate challenge in our effort to fight cancer as a life-threatening disease (Wan et al., 2013). Stage II means the tumor cells start to spread to the nearby lymph nodes (Chiang and Massagué, 2008). These results of the proposed method are highly consistent with the actual biological processes.

To validate the identification of the pre-disease stage, a survival analysis is performed between the samples from different stages (Figures 4D–F). Compared with the samples from the stages in the disease state, the samples from the stages in normal and pre-disease states have higher life expectancy. For BRCA, Figure 4A shows that the ranking score has a sudden increase at stages IB and II, after which the tumor cells spread to the nearby tissue (Chiang and Massagué, 2008). As shown in Figure 4D, a significant difference (*p* = 0.0006) exists between the survival curves of samples from stages I–II and stages IIA–X. Samples from stages I–II have significantly longer survival periods than samples from stage IIA–X. For ESCA, Figure 4B shows that there is a sudden increase at stages IB and II in the ranking score. Figure 4E shows a significant difference between the survival periods of samples in stages I–II and stages IIA–IVB. For READ, as shown in Figure 4C, the drastic increase in the ranking score appears in stages IIB and IIC. Figure 4F shows that there is a significant difference between the survival curves of samples in stages I–IIC and samples in stages III–IVA. These results demonstrate that S-PCR can detect the early warning signals of a critical transition of the biological process of a tumor; that is, the critical transition associated with nearby metastasis at stage II can be identified by the proposed method.

### Functional analysis for influenza infection

To validate the effectiveness of the proposed ranking score, the biological process GO term enrichment analysis and KEGG pathway analysis are carried out for each dataset based on the common candidate signaling genes that appear in more than four symptomatic subjects in datasets GSE30550 and GSE52428. For each individual symptomatic subject in the datasets at the early warning signal appearing time point, the top 5% genes with the largest ranking scores are selected as candidate signaling genes.

**FIGURE 5:**
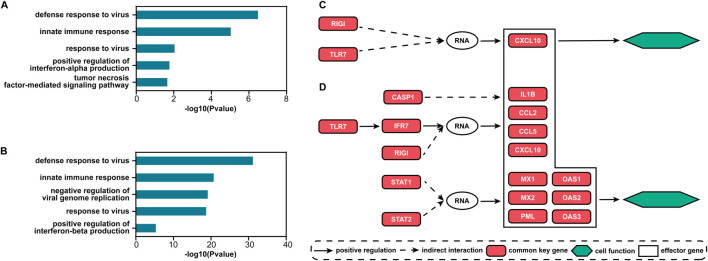
Results of functional analysis for the two influenza infection datasets. Top five GO terms with the smallest FDR-adjusted *p*-values for **(A)** GSE30550 and **(B)** GSE52428. The main direct and indirect interactions between the common and effector signaling genes in the pathway *influenza A* for **(C)** GSE30550 and **(D)** GSE52428.

For the two datasets, there are 70 and 225 common signaling genes (see Supplementary Table S1), respectively. Based on these genes, the top five GO terms with the smallest FDR-adjusted *p*-values of enrichment analysis for each dataset are shown in Figures 5A, B. The biological processes of immunity or defense against the influenza virus including “defense response to virus,” “innate immune response,” and “response to virus” are all ranked in the top three. The other two terms are also highly associated with the process of influenza virus infection. Interferon (IFN)-α can protect against influenza viral infection indirectly by promoting neutrophil respiratory burst responses (Little et al., 1994). Thus, the GO term “positive regulation of interferon-alpha production” may be highly associated with the process of influenza virus infection. Furthermore, in the lung epithelial cells, the tumor necrosis factor alpha can exert powerful anti-influenza virus effects (Seo and Webster, 2002). Therefore, the GO term “tumor necrosis factor-mediated signaling pathway” may also play an important role during influenza virus infection. The GO term “negative regulation of viral genome replication” is also a biological process of defense against the influenza virus. The antiviral effects of IFN-β against influenza virus infections are well recognized (Yoo et al., 2010). So, the GO term “positive regulation of interferon-beta production” is an important biological process during influenza virus infection. All enriched GO terms for influenza virus infection are given in Supplementary Table S2. Clearly, the signaling genes are involved in important biological processes during influenza virus infection. Such results imply that S-PCR can also help understand the biological mechanism of influenza early infection. The common signaling genes can also be seen as the biomarker for disease early diagnosis and potential drug targets against influenza virus infection.

Figures 5C, D show the main direct and indirect interactions between the common and effector signaling genes (Bao et al., 2020) in the pathway *influenza A* for the two influenza infection datasets. These effector genes typically decide cell functional attributes. The functions of the genes that are both common key and effector genes are validated to be highly associated with influenza virus infection. CCL2 and CCL5 encoding proteins are included in the CC subfamily of chemokines, which are a superfamily of secreted proteins involved in immunoregulatory and inflammatory processes, and play pivotal roles in controlling leukocyte recruitment during inflammatory responses (Zlotnik and Yoshie, 2012). CXCL10 and IL1B encoding proteins are the pro-inflammatory cytokines that are involved in a wide variety of processes, and thereby play an important role during viral infections by stimulating the activation and migration of immune cells to the infected sites (Van Damme et al., 1985; Angiolillo et al., 1995). MX1 and MX2 encoding proteins are included in interferon-induced dynamin-like GTPase, which inhibits several different viruses by blocking the early steps of the viral replication cycle (Haller et al., 2015). PML proteins, the key component of subnuclear structures known as PML nuclear bodies (PML-NBs), are involved in the antiviral defense against a variety of DNA and RNA viruses (Scherer and Stamminger, 2016). OAS1, OAS2, and OAS3 encoding enzymes all play critical roles in cellular innate antiviral response (Sarkar et al., 1999; Wickenhagen et al., 2021).

### Functional analysis for cancers

To further validate the effectiveness of the proposed ranking score, the biological process GO term enrichment and KEGG pathway analyses are also carried out for each cancer dataset based on the candidate signaling genes. For each cancer type, the top 5% genes with the largest ranking scores in the predicted pre-disease stage are selected as signaling genes (see Supplementary Table S3).

**FIGURE 6:**
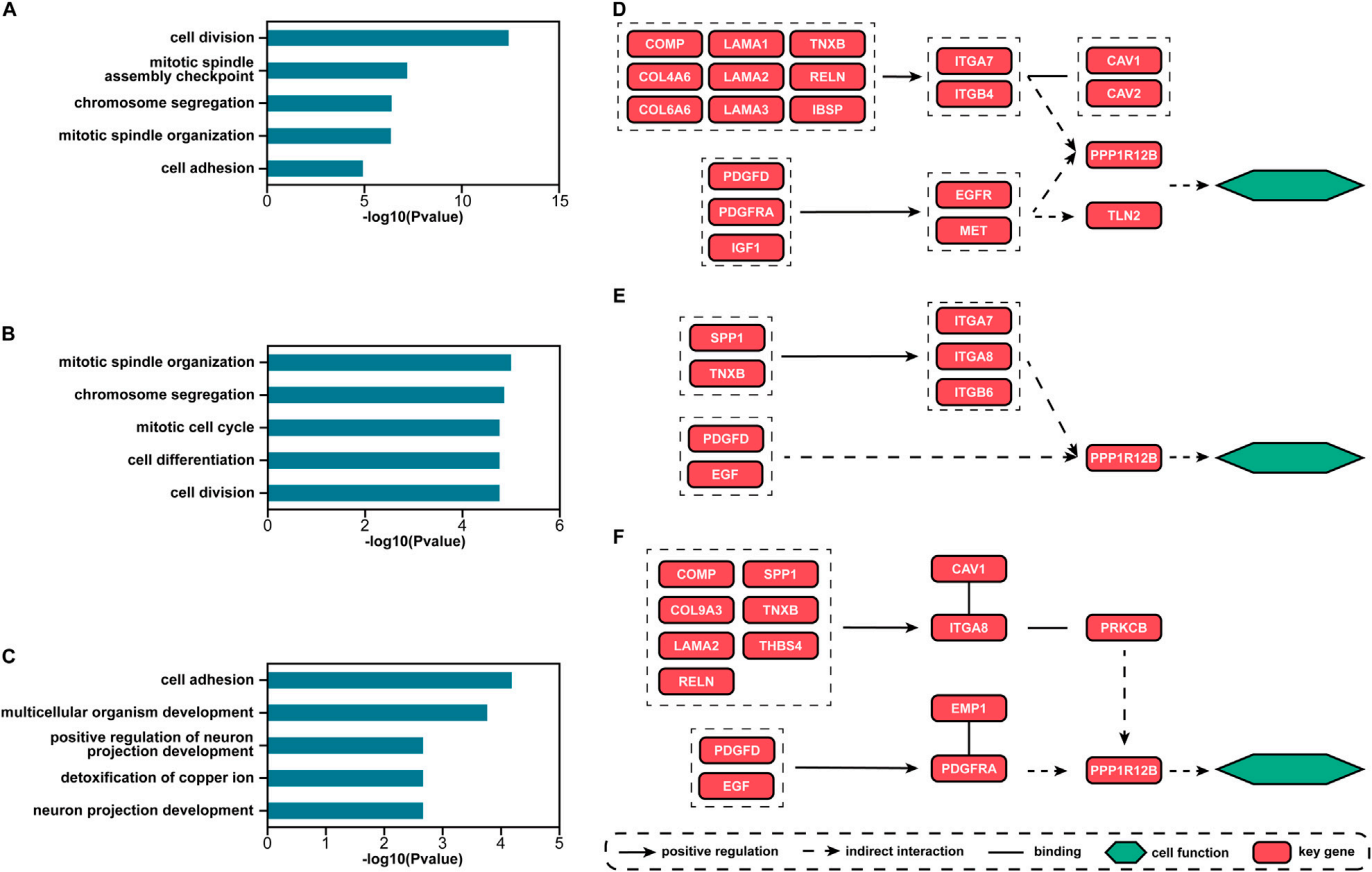
Results of the functional analysis for the three cancer datasets. The top five GO terms with the smallest FDR-adjusted *p*-values for **(A)** BRCA, **(B)** ESCA, and **(C)** READ. The main interactions between the signaling genes in the pathway *Focal adhesion* for **(D)** BRCA, **(E)** ESCA, and **(F)** READ.

The top five GO terms with the smallest FDR-adjusted *p*-values of enrichment analysis for each cancer dataset are shown in Figures 6A–C. All enriched GO terms for cancer metastasis are given in Supplementary Table S4. The dysregulations of these biological process GO terms are highly associated with cancer. For example, the dysregulation of the mitotic spindle assembly checkpoint may cause chromosomal instability, which is a driving force for cancer development (Yu et al., 2022). Tumorigenesis in humans is a multistep process, which can reflect genetic alterations that drive the progressive transformation of normal cells into highly malignant derivatives, including cell differentiation and cell division (Hanahan and Weinberg, 2011). Gross chromosomal aberrations are usually lethal but can cause cancer (Curtis et al., 2020). Thus, the GO terms “cell division,” “cell differentiation,” “mitotic cell cycle,” “mitotic spindle assembly checkpoint,” “mitotic spindle organization,” and “chromosome segregation” are highly related to cancers. Many studies have elucidated that cellular adhesion processes are highly associated with cancer malignant transformation and metastasis (Janiszewska et al., 2020). Therefore, the GO term “cell adhesion” is highly associated with cancers. Cancer can be understood as a failure of multicellular systems to suppress somatic evolution (Nedelcu, 2020). So, the GO term “multicellular organism development” is related to cancers. The GO terms “neuron projection development” and “positive regulation of neuron projection development” are highly related to cancers because nerves are important pathological elements of the microenvironment of tumors (Wang et al., 2021). Alterations in Cu homeostasis may promote tumor growth or invasiveness, and may even confer resistance to cancer treatments (Wang et al., 2021). Thus, the dysregulation of the GO term “detoxification of copper ion” may be associated with cancers. Clearly, the signaling genes are involved in important biological processes to define the influenza virus infection. Such results imply that S-PCR can also help understand the biological mechanism of early influenza infection. The signaling genes can also be seen as the biomarker for disease early diagnosis and potential drug targets.

Ample evidence has indicated that the *focal adhesion* signaling pathway can stimulate metastasis through its regulation of cell migration, invasion, and angiogenesis (Owen et al., 1999; Hauck et al., 2002; Luo and Guan, 2010). Thus, the *focal adhesion* pathway in the KEGG database is used to perform the KEGG pathway analysis for the three cancer datasets. Figures 6D–F show the interactions between signaling genes for each cancer type. In the KEGG database, the *focal adhesion* pathway mainly involves three cell functions: cell motility, cell proliferation, and cell survival. As shown in the figures, these signaling genes selected by the three cancer datasets in the *focal adhesion* pathway are all highly associated with cell function and cell motility, which is highly associated with tumor metastasis (Stuelten et al., 2018; Palmer et al., 2011). In general, the above results suggest that in cancer progression, abnormal cell motility would cause the uncontrolled progression of the cell cycle, ultimately causing further malignant metastasis.

### Time complexity and running time comparison

The previous results of the simulated and five real datasets validated the good performance of the proposed method S-PCR in accurately identifying the pre-disease state. The results of the functional analysis also demonstrated the effectiveness of the ranking score used in this computational approach. In addition to these, profiting from the use of gene expression ranking, the time complexity and running time of S-PCR are greatly reduced compared to other methods.

Network and entropy-based methods always need to calculate the entropy based on complex biological networks. Furthermore, gene expression distribution-based methods need to fit the distribution comparison for thousands of genes. Thus, previous single-sample suitable methods are always complex. S-PCR only needs to acquire the expression ranking of each time point and then calculate the change in such ranking and expression value by matrix operation, which can omit such limitations. So, compared to other methods, S-PCR is a simple method. To validate the simplicity of S-PCR, the time complexity of the proposed method is compared to other methods, SLE (Liu et al., 2020) and sJSD (Yan et al., 2021), which is the classical method for the methods based on the network and gene expression distribution, respectively. If we use genomic data including total *n* genes to perform the three methods, the time complexity analysis of the three methods is as follows. The main step in S-PCR is the calculation of gene expression ranking using a fast sort algorithm. Thus, the time complexity of S-PCR is O(nlogn). The main step of the SLE method is the calculation of Pearson’s correlation coefficient between the whole n genes. Thus, the time complexity of SLE is O(n^2^). For sJSD, the expression curve and probability for each gene should be calculated before calculating Jensen–Shannon divergence for each gene using *m* normal or case samples. Therefore, the time complexity of sJSD is also O(n^2^).

**FIGURE 7:**
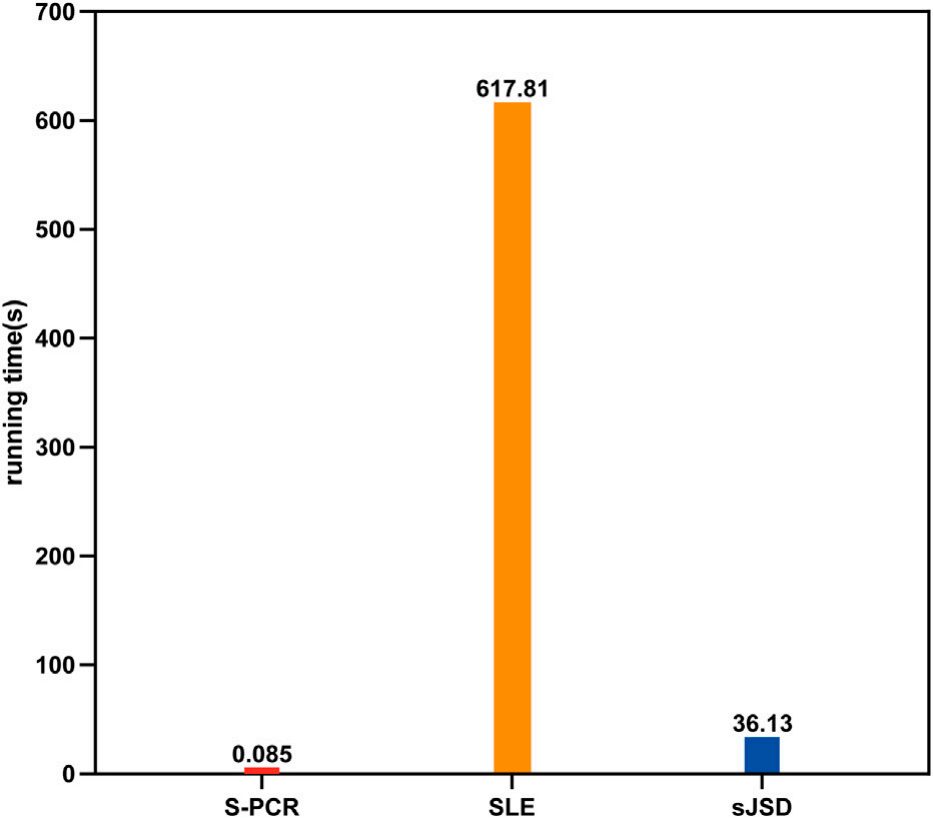
Average running time of three methods for each symptomatic subject in GSE30550.

Extrapolating from the result of time complexity comparison, our method should run much faster than SLE and sJSD with the same computational machine. To validate such results, the average running time for each symptomatic subject in GSE30550 of the proposed method is compared to that of the other two methods. S-PCR is performed using R 4.0.3. SLE and sJSD are performed using MATLAB 2018b. All numerical experiments are done on a Windows computer with eight Core 2.3 GHz processors (Intel Core i7-10875H CPU) and 16 GB of physical memory. As shown in Figure 7, our method is implemented much faster than SLE and sJSD on the same computer. Therefore, the S-PCR method can better simplify the processes of personalized early disease prevention and promote its development.

### Advantage of using gene expression ranking in S-PCR

**FIGURE 8:**
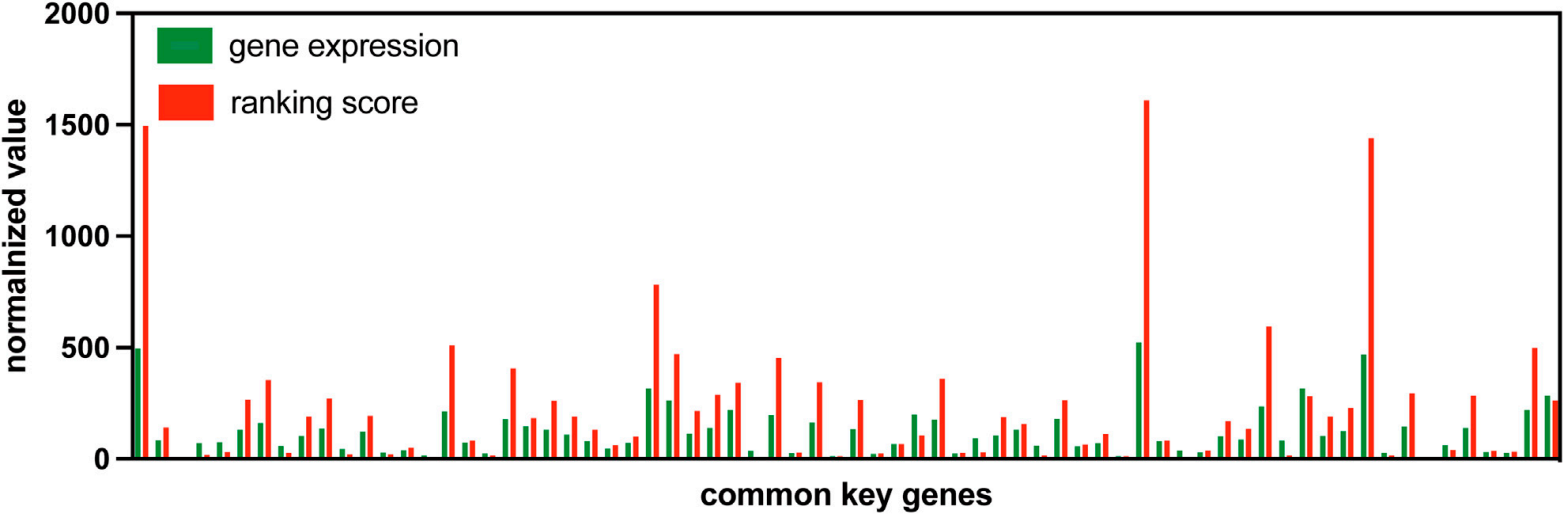
Normalized gene expression values and rank scores of 70 common signaling genes for subject 01 in GSE30550.

By using the change in gene expression ranking, S-PCR can amplify the early warning signal just like DNA PCR amplification techniques. To validate this advantage of gene expression ranking change, the gene expression value is compared with the ranking score. Figure 8 shows the normalized gene expression values and ranking scores of symptomatic subject 01 across 70 common signaling genes for dataset GSE30550.

The gene expression value and ranking score are all normalized in a similar way to make the comparison at the same level. For a common signaling gene $k_{J}$ , the normalized gene expression value and ranking score are normalized by the minimum value across all common signaling genes, as Formula 7 shown.

$$\Delta V_{j}=\frac{V_{j}}{\min\left(V_{1},V_{2},\cdots ,V_{70}\right)},$$ (7)

where $\Delta V_{j}$ is the normalized gene expression value or ranking score of the common signaling gene $k_{j}$ . $V_{j}$ is the original gene expression value or ranking score of the gene $k_{j}$ . $V_{1},V_{2},\cdots ,V_{70}$ are the gene expression values or ranking scores of the 70 common signaling genes, respectively.

As the figure shows, the normalized ranking scores for most common signaling genes are much more than their normalized gene expression values. Such a result indicates that the usage of gene expression ranking can amplify the early warning signal.

## Discussion

Detecting the pre-disease state is of great importance to signal the upcoming disease, which is helpful for the development of disease prevention and ultra-early precision treatment. Chen et al. proposed the DNB method to predict the pre-disease state (Chen et al., 2012; Liu et al., 2012). However, this method needs to select a small group of molecules using three measurements, including the calculation of Pearson’s correlation coefficient and standard deviation, which need multiple samples at each time point. In clinical practice, only one sample can be gained at each time point. Thus, the DNB method is unsuitable for clinical single-sample datasets. In this study, we propose a novel pre-disease state prediction method based on the ranking change in gene expression following the DNB theory, which is suitable for single-sample datasets, called S-PCR. A simulated dataset, two influenza virus infection datasets, and three TCGA cancer datasets are used to measure the performance of the proposed method. The results are consistent with those of the clinical and experimental observations. For the six datasets, only one symptomatic subject from dataset GSE52428 could not be found during the pre-disease state. Furthermore, the signaling genes detected by S-PCR for the five real disease datasets are always highly related to the pre-disease state-related biological processes and cell functions. Thus, the accuracy of S-PCR is competitive. Furthermore, the time efficiency of S-PCR is also validated by comparing with other methods. Furthermore, using the gene expression ranking change in S-PCR can help us better identify gene expression fluctuating signals at the pre-disease state.

Additionally, S-PCR is a gene expression fluctuating signal-sensitive approach. The pre-disease state is always defined as the limit of the normal state. Thus, the expression value of a gene at the pre-disease state may have little/no difference from that at the normal state. However, the expression ranking of the gene may have a bigger change from the pre-disease to the normal state. On the other hand, if the expression values of whole genes in the system are all changed at the same time, the gene expression rankings of these genes may have little/no changes. From these aspects, the proposed S-PCR can remove some noises in gene expression data and is a gene expression-fluctuating signal-sensitive approach.

However, we should point out that using gene expression ranking may introduce some noises for that there are no interactions between some genes with huge changes in the expression ranking. In the future, there are two ways to solve this problem. First, the credible small-scale functional gene regulatory networks or relevant key regulatory targets, which are identified to be highly associated with the initial progression of complex diseases, can be introduced instead of the whole genome (Zhenshen et al., 2023; Jin et al., 2023). This is because the genes in these sets always interact strongly with each other. Second, energy landscape theory can be used to amplify abnormal signals and minimize the impact of noise. Thus, the expression ranking change can be further combined with the energy landscape theory (Jin et al., 2024).

## Conclusion

In this paper, a method called S-PCR to rapidly identify the pre-disease state of complex diseases for a single sample is presented. S-PCR obtains a set of DNBs by using the change in gene expression ranking. Benefiting from the use of gene expression ranking, the calculation performed using the S-PCR method takes much less time than that performed using other methods. Therefore, S-PCR can accurately identify the pre-disease state in less time than other methods. Hence, the S-PCR method has great potential in clinical early prevention medicine.

## Data Availability

The original contributions presented in the study are included in the article/Supplementary Material; further inquiries can be directed to the corresponding authors.

## References

[B1] Achiron A. Grotto I. Balicer R. Magalashvili D. Feldman A. Gurevich M. (2010). Microarray analysis identifies altered regulation of nuclear receptor family members in the pre-disease state of multiple sclerosis. Neurobiol. Dis. 38, 201–209. 10.1016/j.nbd.2009.12.029 20079437

[B2] Angiolillo A. L. Sgadari C. Taub D. D. Liao F. Farber J. M. Maheshwari S. (1995). Human interferon-inducible protein 10 is a potent inhibitor of angiogenesis *in vivo* . J. Exp. Med. 182, 155–162. 10.1084/jem.182.1.155 7540647 PMC2192108

[B3] Bao Z. Zhang B. Li L. Ge Q. Gu W. Bai Y. (2020). Identifying disease-associated signaling pathways through a novel effector gene analysis. PeerJ 8, e9695. 10.7717/peerj.9695 32864216 PMC7430270

[B4] Bao Z. Zheng Y. Li X. Huo Y. Zhao G. Zhang F. (2022). A simple pre-disease state prediction method based on variations of gene vector features. Comput. Biol. Med. 148, 105890. 10.1016/j.compbiomed.2022.105890 35940162

[B5] Chen L. Liu R. Liu Z. P. Li M. Aihara K. (2012). Detecting early-warning signals for sudden deterioration of complex diseases by dynamical network biomarkers. Sci. Rep. 2, 342. 10.1038/srep00342 22461973 PMC3314989

[B6] Chiang A. C. Massagué J. (2008). Molecular basis of metastasis. N. Engl. J. Med. 359, 2814–2823. 10.1056/NEJMra0805239 19109576 PMC4189180

[B7] Curtis N. L. Ruda G. F. Brennan P. Bolanos-Garcia V. M. (2020). Deregulation of chromosome segregation and cancer. Annu. Rev. Cancer Biol. 4, 257–278. 10.1146/annurev-cancerbio-030419-033541

[B8] Haller O. Staeheli P. Schwemmle M. Kochs G. (2015). Mx GTPases: dynamin-like antiviral machines of innate immunity. Trends Microbiol. 23, 154–163. 10.1016/j.tim.2014.12.003 25572883

[B9] Han C. Zhong J. Hu J. Liu H. Liu R. Ling F. (2020). Single-sample node entropy for molecular transition in pre-deterioration stage of cancer. Front. Bioeng. Biotechnol. 8, 809. 10.3389/fbioe.2020.00809 32766227 PMC7381145

[B10] Hanahan D. Weinberg R. A. (2011). Hallmarks of cancer: the next generation. Cell 144, 646–674. 10.1016/j.cell.2011.02.013 21376230

[B11] Hauck C. R. Hsia D. A. Puente X. S. Cheresh D. A. Schlaepfer D. D. (2002). FRNK blocks v-Src-stimulated invasion and experimental metastases without effects on cell motility or growth. Embo J. 21, 6289–6302. 10.1093/emboj/cdf631 12456636 PMC136935

[B12] Hong R. Tong Y. Liu H. Chen P. Liu R. (2024). Edge-based relative entropy as a sensitive indicator of critical transitions in biological systems. J. Transl. Med. 22, 333. 10.1186/s12967-024-05145-3 38576021 PMC10996174

[B13] Huang Y. Zaas A. K. Rao A. Dobigeon N. Woolf P. J. Veldman T. (2011). Temporal dynamics of host molecular responses differentiate symptomatic and asymptomatic influenza a infection. PLoS Genet. 7, e1002234. 10.1371/journal.pgen.1002234 21901105 PMC3161909

[B14] Huang da W. Sherman B. T. Lempicki R. A. (2009). Systematic and integrative analysis of large gene lists using DAVID bioinformatics resources. Nat. Protoc. 4, 44–57. 10.1038/nprot.2008.211 19131956

[B15] Huo Y. Li C. Li Y. Li X. Xu P. Bao Z. (2023). Detecting early-warning signals for influenza by dysregulated dynamic network biomarkers. Brief. Funct. Genomics 22, 366–374. 10.1093/bfgp/elad006 36787234

[B16] Huo Y. Zhao G. Ruan L. Xu P. Fang G. Zhang F. (2022). Detect the early-warning signals of diseases based on signaling pathway perturbations on a single sample. BMC Bioinforma. 22, 367. 10.1186/s12859-021-04286-2 PMC8772045 35045824

[B17] Janiszewska M. Primi M. C. Izard T. (2020). Cell adhesion in cancer: beyond the migration of single cells. J. Biol. Chem. 295, 2495–2505. 10.1074/jbc.REV119.007759 31937589 PMC7039572

[B18] Jin J. Xu F. Liu J. L. Xiang Yao C. Shuai J. (2023). Biphasic amplitude oscillator characterized by distinct dynamics of trough and crest. Phys. Rev. E 108, 064412. 10.1103/PhysRevE.108.064412 38243441

[B19] Jin J. Xu F. Liu Z. Shuai J. (2024). Quantifying the underlying landscape, entropy production and biological path of the cell fate decision between apoptosis and pyroptosis. Chaos, Solit. and Fractals 178, 114328. 10.1016/j.chaos.2023.114328

[B20] Kanehisa M. Furumichi M. Sato Y. Matsuura Y. Ishiguro-Watanabe M. (2024). KEGG: biological systems database as a model of the real world. Nucleic Acids Res., gkae909. 10.1093/nar/gkae909 39417505 PMC11701520

[B21] Li Y. Jin S. Lei L. Pan Z. Zou X. (2015). Deciphering deterioration mechanisms of complex diseases based on the construction of dynamic networks and systems analysis. Sci. Rep. 5, 9283. 10.1038/srep09283 25788156 PMC4365388

[B22] Little R. White M. R. Hartshorn K. L. (1994). Interferon-alpha enhances neutrophil respiratory burst responses to stimulation with influenza A virus and FMLP. J. Infect. Dis. 170, 802–810. 10.1093/infdis/170.4.802 7930721

[B23] Liu R. Chen P. Chen L. (2020). Single-sample landscape entropy reveals the imminent phase transition during disease progression. Bioinformatics 36, 1522–1532. 10.1093/bioinformatics/btz758 31598632

[B24] Liu R. Li M. Liu Z. P. Wu J. Chen L. Aihara K. (2012). Identifying critical transitions and their leading biomolecular networks in complex diseases. Sentific Rep. 2, 813. 10.1038/srep00813 PMC3517980 23230504

[B25] Liu R. Yu X. Liu X. Xu D. Aihara K. Chen L. (2014). Identifying critical transitions of complex diseases based on a single sample. Bioinformatics 30, 1579–1586. 10.1093/bioinformatics/btu084 24519381

[B26] Luo M. Guan J. L. (2010). Focal adhesion kinase: a prominent determinant in breast cancer initiation, progression and metastasis. Cancer Lett. 289, 127–139. 10.1016/j.canlet.2009.07.005 19643531 PMC2854647

[B27] McSharry P. E. Smith L. A. Tarassenko L. (2003). Prediction of epileptic seizures: are nonlinear methods relevant?. Nat. Med. 9, 241–242. 10.1038/nm0303-241 12612550

[B28] Nedelcu A. M. (2020). The evolution of multicellularity and cancer: views and paradigms. Biochem. Soc. Trans. 48, 1505–1518. 10.1042/BST20190992 32677677

[B29] Owen J. D. Ruest P. J. Fry D. W. Hanks S. K. (1999). Induced focal adhesion kinase (FAK) expression in FAK-null cells enhances cell spreading and migration requiring both auto- and activation loop phosphorylation sites and inhibits adhesion-dependent tyrosine phosphorylation of Pyk2. Mol. Cell Biol. 19, 4806–4818. 10.1128/MCB.19.7.4806 10373530 PMC84279

[B30] Palmer T. D. Ashby W. J. Lewis J. D. Zijlstra A. (2011). Targeting tumor cell motility to prevent metastasis. Adv. Drug Deliv. Rev. 63, 568–581. 10.1016/j.addr.2011.04.008 21664937 PMC3132821

[B31] Sarkar S. N. Ghosh A. Wang H. W. Sung S. S. Sen G. C. (1999). The nature of the catalytic domain of 2'-5'-oligoadenylate synthetases. J. Biol. Chem. 274, 25535–25542. 10.1074/jbc.274.36.25535 10464285

[B32] Scherer M. Stamminger T. (2016). Emerging role of PML nuclear bodies in innate immune signaling. J. Virol. 90, 5850–5854. 10.1128/JVI.01979-15 27053550 PMC4907236

[B33] Seo S. H. Webster R. G. (2002). Tumor necrosis factor alpha exerts powerful anti-influenza virus effects in lung epithelial cells. J. virology 76, 1071–1076. 10.1128/jvi.76.3.1071-1076.2002 11773383 PMC135862

[B34] Sherman B. T. Hao M. Qiu J. Jiao X. Baseler M. W. Lane H. C. (2022). DAVID: a web server for functional enrichment analysis and functional annotation of gene lists (2021 update). Nucleic Acids Res. 50, W216–w221. 10.1093/nar/gkac194 35325185 PMC9252805

[B35] Srinivasan B. (2022). A guide to the Michaelis-Menten equation: steady state and beyond. Febs J. 289, 6086–6098. 10.1111/febs.16124 34270860

[B36] Stuelten C. H. Parent C. A. Montell D. J. (2018). Cell motility in cancer invasion and metastasis: insights from simple model organisms. Nat. Rev. Cancer 18, 296–312. 10.1038/nrc.2018.15 29546880 PMC6790333

[B37] Subramanian A. Tamayo P. Mootha V. K. Mukherjee S. Ebert B. L. Gillette M. A. (2005). Gene set enrichment analysis: a knowledge-based approach for interpreting genome-wide expression profiles. Proc. Natl. Acad. Sci. 102, 15545–15550. 10.1073/pnas.0506580102 16199517 PMC1239896

[B38] Van Damme J. De Ley M. Opdenakker G. Billiau A. De Somer P. Van Beeumen J. (1985). Homogeneous interferon-inducing 22K factor is related to endogenous pyrogen and interleukin-1. Nature 314, 266–268. 10.1038/314266a0 3920526

[B39] Venegas J. G. Winkler T. Musch G. Vidal Melo M. F. Layfield D. Tgavalekos N. (2005). Self-organized patchiness in asthma as a prelude to catastrophic shifts. Nature 434, 777–782. 10.1038/nature03490 15772676

[B40] Wan L. Pantel K. Kang Y. (2013). Tumor metastasis: moving new biological insights into the clinic. Nat. Med. 19, 1450–1464. 10.1038/nm.3391 24202397

[B41] Wang H. Zheng Q. Lu Z. Wang L. Ding L. Xia L. (2021). Role of the nervous system in cancers: a review. Cell Death Discov. 7, 76. 10.1038/s41420-021-00450-y 33846291 PMC8041826

[B42] Wickenhagen A. Sugrue E. Lytras S. Kuchi S. Noerenberg M. Turnbull M. L. (2021). A prenylated dsRNA sensor protects against severe COVID-19. Science 374, eabj3624. 10.1126/science.abj3624 34581622 PMC7612834

[B43] Woods C. W. McClain M. T. Chen M. Zaas A. K. Nicholson B. P. Varkey J. (2013). A host transcriptional signature for presymptomatic detection of infection in humans exposed to influenza H1N1 or H3N2. PLoS One 8, e52198. 10.1371/journal.pone.0052198 23326326 PMC3541408

[B44] Yan J. Li P. Gao R. Li Y. Chen L. (2021). Identifying critical states of complex diseases by single-sample jensen-shannon divergence. Front. Oncol. 11, 684781. 10.3389/fonc.2021.684781 34150649 PMC8212786

[B45] Yoo J. K. Baker D. P. Fish E. N. (2010). Interferon-β modulates type 1 immunity during influenza virus infection. Antivir. Res. 88, 64–71. 10.1016/j.antiviral.2010.07.006 20659503

[B46] Yu L. Lang Y. Hsu C. C. Chen W. M. Chiang J. C. Hsieh J. T. (2022). Mitotic phosphorylation of tumor suppressor DAB2IP maintains spindle assembly checkpoint and chromosomal stability through activating PLK1-Mps1 signal pathway and stabilizing mitotic checkpoint complex. Oncogene 41, 489–501. 10.1038/s41388-021-02106-8 34775484 PMC8782720

[B47] Yu X. Zhang J. Sun S. Zhou X. Zeng T. Chen L. (2017). Individual-specific edge-network analysis for disease prediction. Nucleic Acids Res. 45, e170. 10.1093/nar/gkx787 28981699 PMC5714249

[B48] Zhenshen B. Minzhen L. Wanqi D. Huo Y. Li X. Xu P. (2023). A pan-cancer analysis reveals the tissue specificity and prognostic impact of angiogenesis-associated genes in human cancers. Curr. Bioinforma. 18, 670–679. 10.2174/1574893618666230518163353

[B49] Zhong J. Ding D. Liu J. Liu R. Chen P. (2023). SPNE: sample-perturbed network entropy for revealing critical states of complex biological systems. Brief. Bioinform 24, bbad028. 10.1093/bib/bbad028 36705581

[B50] Zhong J. Han C. Wang Y. Chen P. Liu R. (2022). Identifying the critical state of complex biological systems by the directed-network rank score method. Bioinformatics 38, 5398–5405. 10.1093/bioinformatics/btac707 36282843 PMC9750123

[B51] Zhong J. Liu R. Chen P. (2020). Identifying critical state of complex diseases by single-sample Kullback-Leibler divergence. BMC genomics 21, 87. 10.1186/s12864-020-6490-7 31992202 PMC6988219

[B52] Zlotnik A. Yoshie O. (2012). The chemokine superfamily revisited. Immunity 36, 705–716. 10.1016/j.immuni.2012.05.008 22633458 PMC3396424

